# Ubiquitin C-terminal hydrolase L1 (UCH-L1): structure, distribution and roles in brain function and dysfunction

**DOI:** 10.1042/BCJ20160082

**Published:** 2016-08-11

**Authors:** Paul Bishop, Dan Rocca, Jeremy M. Henley

**Affiliations:** * School of Biochemistry, Centre for Synaptic Plasticity, Biomedical Sciences Building, University of Bristol, Bristol BS8 1TD, U.K.

**Keywords:** axon, neurites, ubiquitin C-terminal hydrolase L1 (UCH-L1), ubiquitin ligases, ubiquitin proteasome system

## Abstract

Ubiquitin C-terminal hydrolase L1 (UCH-L1) is an extremely abundant protein in the brain where, remarkably, it is estimated to make up 1–5% of total neuronal protein. Although it comprises only 223 amino acids it has one of the most complicated 3D knotted structures yet discovered. Beyond its expression in neurons UCH-L1 has only very limited expression in other healthy tissues but it is highly expressed in several forms of cancer. Although UCH-L1 is classed as a deubiquitinating enzyme (DUB) the direct functions of UCH-L1 remain enigmatic and a wide array of alternative functions has been proposed. UCH-L1 is not essential for neuronal development but it is absolutely required for the maintenance of axonal integrity and UCH-L1 dysfunction is implicated in neurodegenerative disease. Here we review the properties of UCH-L1, and how understanding its complex structure can provide new insights into its roles in neuronal function and pathology.

## INTRODUCTION

The identification and safe destruction of unwanted, misfolded or aggregated proteins is essential for cell viability. The complexity and sophistication of neuronal architecture and signalling pathways make them especially vulnerable to protein aggregation and failure to adequately destroy proteins underlies multiple neuropathologies [1].

### The ubiquitin system

Ubiquitin is a highly conserved 76-amino acid protein that can be conjugated, either singly or as polyubiquitin chains, to residues in a target protein to alter its function and/or fate. Lysine is the most common residue to undergo ubiquitination, although non-canonical serine, threonine and cysteine side chains, as well as the N-terminal amino group, can also be modified [2]. The ubiquitin system is central to the regulation of almost all cellular processes because it controls protein activity and abundance [3]. Substrate proteins tagged with Lys^48^-linked polyubiquitin chains enter the ubiquitin-proteasome system (UPS), which mediates their degradation via the 26S proteasome [4–6]. Ubiquitinated proteins can also be targeted for lysosomal degradation. Monoubiquitination can be a tag for recruiting substrates into the lysosomal pathway via the ESCRT complex [7].

In addition to protein degradation, ubiquitination can mediate a wide variety of cellular events, ranging from protein membrane trafficking and endocytosis to DNA repair [8]. In neurons, ubiquitination plays a major role in regulating neuronal development, function and pathology [9]. For example, enhancing or reducing synaptic activity reciprocally regulates the properties, localization and abundance of many proteins [10,11], but how the ubiquitin system itself is regulated, and the consequences of its function and dysfunction on individual synaptic proteins and signalling networks remain largely unknown.

### UCH class of DUBs

Ubiquitin is removed from substrate proteins by deubiquitinases (DUBs). There are ∼90 DUBs in the human genome, of which the ubiquitin C-terminal hydrolase (UCH) subgroup has four members. Each UCH contains an N-terminal C12 peptidase domain formed from a knotted peptide backbone, a C-terminal extension and an unstructured loop that regulates substrate access to the catalytic site. UCH DUBs are implicated in a diverse range of pathways (Table 1). *In vitro* experiments suggest that they cleave C-terminal peptide adducts as well as N-terminally conjugated ubiquitin from substrate proteins [12] and it has also been proposed that UCHs can deubiquitinate small nucleophiles, such as glutathione, which become aberrantly modified in the cytoplasm [13–15].

**Table 1:** Core characteristics of the Ub C-terminal hydrolase (UCH) family of deubiquitinating enzymes (DUBs)

UCH DUB	Length	C-terminal extension	Function	References
UCH-L1	223 aa	Small, unstructured	Currently unclear. Abundantly expressed in neurons, testes and ovaries	[16,45]
UCH-L3	233 aa	Small, unstructured	Shares 52% sequence homology with UCH-L1 but is more widely expressed throughout mammalian tissues. Hydrolyses the disease-associated frame-shifted Ubb+1 ubiquitin molecule	[46,110]
UCH-L5 (UCH37)	329 aa	Fibrous domain that interacts with the Rpn13 subunit of the 26S proteasome	The only member of the UCH class known to play a direct role in proteasomal function, responsible for Lys^48^ ubiquitin isopeptidase activity to recycle ubiquitin from proteasomal degradation	[48,49]
BAP1	729 aa	Long extension contains a nuclear localization signal	Plays a role in histone ubiquitination, chromatin remodelling and transcriptional regulation as well as inhibiting activity of BRCA1	[111,112]

### UCH-L1 distribution

The tissue distribution of ubiquitin C-terminal hydrolase L1 (UCH-L1) is predominantly within the brain where it can make up to 5% of total neuronal protein [16,17], but it is also present at much lower levels in the gonads and is weakly expressed in some cells under specialized conditions, such as human fibroblasts during wound healing, and in some clonal cell cultures [18,19]. Intriguingly, it is also present in cancerous cells originating from tissues that do not normally express UCH-L1, including pancreatic cancer, colorectal cancer and invasive breast cancer [20–22].

At a cellular level UCH-L1 exhibits strong, uniform cytoplasmic staining in neurons throughout the brain [16] and is also present in large sensory and motor neurons [23]. Consistent with this, a transgenic mouse in which the UCH-L1 promoter and 5′UTR were used to drive expression of an eGFP displays robust fluorescence in subsets of cortical neurons and corticospinal motor neurons [24]. This abundance of UCH-L1 in neurons, coupled with its restricted distribution in other tissues, has led to the clinical use of UCH-L1 as a neuron-specific biomarker for severe brain trauma [25,26].

### Membrane association

Although UCH-L1 is mainly cytosolic various reports have suggested that between 20 and 50% can be membrane associated [25,27,28]. Interestingly, however, subcellular fractionation of clonal cell lines did not detect membrane associated UCH-L1 in COS7 or HEK293 cells whereas it was present in cultured rat neurons and adult brain [29]. UCH-L1 lacks obvious lipid interaction domains but since many DUBs can operate as part of larger protein complexes that may well be membrane bound [30], it is likely that UCH-L1 membrane association occurs indirectly via such macromolecular complexes in neurons [29].

## UCH-L1 STRUCTURE

### Knotted backbone

**Figure 1:**
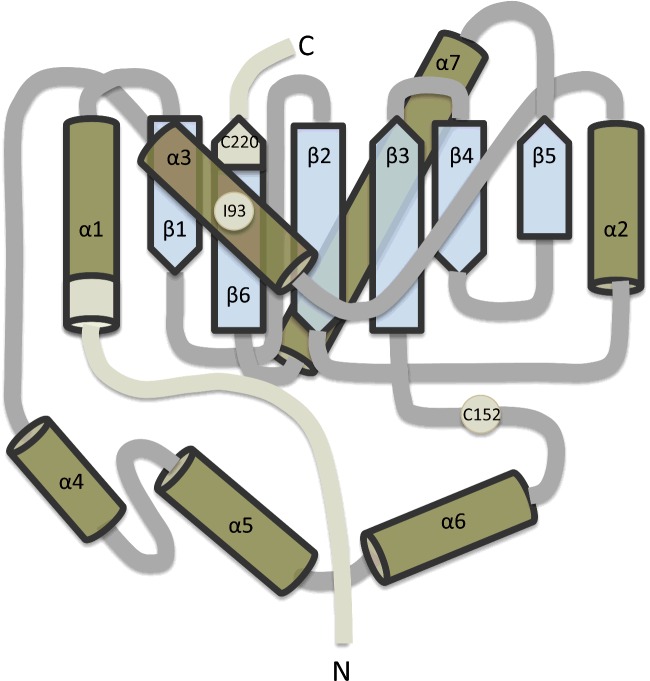
Schematic of UCH-L1 structure Schematic illustrating the α-helical and β-strand structure of UCH-L1. The residues 1–11 at the N-terminus, 220–223 at the C-terminus and residues Ile^93^ and Cys^152^ are highlighted. It has been proposed that modification at these points can affect the hydrophobic core of β-strands that are otherwise protected from solution.

**Figure 2:**
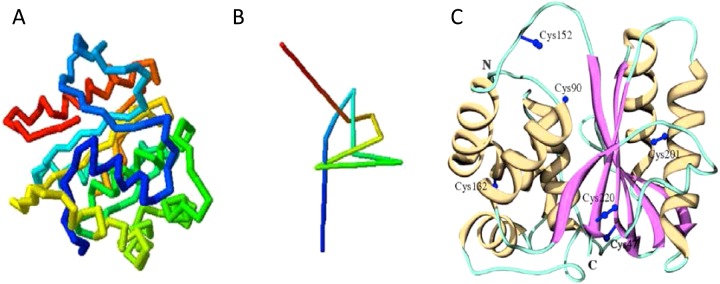
UCH-L1 knotted backbone (**A**) Schematic representation of the peptide backbone structure of UCH-L1. (**B**) A simplified schematic of UCH-L1 backbone knot. Schematics taken from [16]: Day, I.N. and Thompson, R.J. (2010) UCHL1 (PGP 9.5): neuronal biomarker and ubiquitin system protein. Prog. Neurobiol. **90**, 327–362, with permission. (**C**) Crystal structure of UCH-L1 secondary structure highlighting the two ‘lobes’ of α-helices surrounding the β-strands in the hydrophobic core. The location of the six cysteine residues are in blue. The location of Cys^90^ in the catalytic triad and Cys^152^ in the short loop covering the active site can be observed. Schematic from [36]: Koharudin, L.M., Liu, H., Di Maio, R., Kodali, R.B., Graham, S.H. and Gronenborn, A.M. (2010) Cyclopentenone prostaglandin-induced unfolding and aggregation of the Parkinson disease-associated UCH-L1. Proc. Natl. Acad. Sci. U.S.A. **107**, 6835–6840, with permission.

UCH-L1 is a globular protein comprising a conserved peptidase C12 superfamily catalytic domain with very short N- and C-terminal extensions [31] (Figure 1). There are five crossings of the peptidase C12 polypeptide backbone forming a ‘5_2_’ or ‘Gordian’ knot (Figure 2). This knot has been described as the most complicated eukaryotic protein structure discovered to date [32]. The overall 3D structure results in two ‘lobes’ of α-helices surrounding a tightly-packed conserved hydrophobic core of β-strands [31]. Based on the role of UCH-L5 in recycling ubiquitin from proteasomal degradation, it is believed that the knotted backbone evolved to protect UCH class DUBs from unintended proteasomal unfolding and degradation [33].

### Hydrophobic core

**Figure 3:**
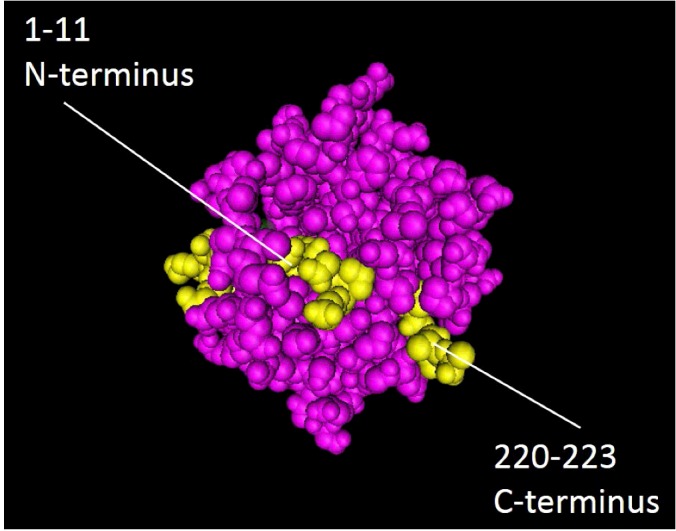
Folding arrangement of N- and C-terminal domains Positions of the N-terminal (residues 1–11) and C-terminal (residues 220–223) domains. Residues indicated in yellow illustrate how the N- and C-terminal sequences penetrate into the hydrophobic core of the protein and how deletion of either of these regions results in loss of solubility and misfolding (diagram drawn using Cn3D http://www.ncbi.nlm.nih.gov/Structure/CN3D/cn3d.shtml).

UCH-L1 unfolds with three populated states, transitioning from folded to fully denatured via an intermediate stage where the α-helices have unfolded but the central hydrophobic core of β-strands remains intact [34]. *In silico* simulations and *in vitro* mutagenic studies indicate that removal of relatively few amino acids from either the N- or C-terminus can destabilize the 3D structure, resulting in unfolding and loss of solubility consistent with protein aggregation [29,32], likely through exposure of this hydrophobic core. Removal of eleven amino acids from the N-terminus is sufficient for the protein to lose affinity for ubiquitin and ultimately leads to the formation of insoluble aggregates [35]. This region includes a portion of the α1 helix, which penetrates into the core of the protein and contacts the β1-strand (Figure 3). Similarly, the loss of just four amino acids from the C-terminus, which includes a portion of the β6-strand, is sufficient to make the protein insoluble and abolish binding to ubiquitin-substrates [29]. Both truncations result in exposure of the hydrophobic core β-sheets to the cytosol causing a loss of conformational integrity, insolubility and neuronal death [36,37]. Rather than a loss of function, as observed in UCH-L1-deficient animal models (see below), UCH-L1 unfolding leads to a toxic gain-of-function. This is most likely due to the exposure of previously hidden hydrophobic regions causing aberrant interactions with other proteins and cellular membranes, as occurs for other misfolded proteins [38–40].

### Active/inactive conformations

**Figure 4:**
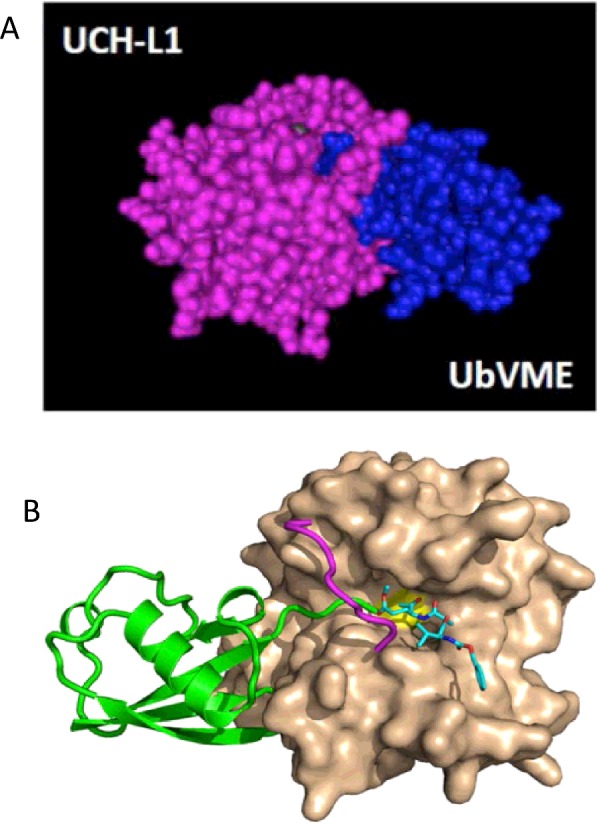
Short loop covering UCH-L1 active site (**A**) UCH-L1 covalently binds ubiquitin substrate. Space-filling molecular model showing UCH-L1 (purple) covalently bound to UbVME (blue), generated using Cn3D software and based on PDB crystal structure 3KW5. (**B**) Crystal structure shows UCH-L1 (beige) bound to UbVME substrate (green). It is believed that the short loop (purple) covering the active site (yellow) limits access to short unfolded peptides only (generated by and used with permission from Chittaranjan Daas, Purdue University).

Many DUBs exist in an ‘inactive’ state that requires additional protein–protein interactions to adopt an ‘active’ conformation, which protects against aberrant hydrolytic activity [41]. In the unbound (*apo*) state, the geometry of the aspartate, histidine and cysteine residues that form the catalytic triad of the active site for hydrolysis is distorted, making the enzyme non-functional, with the His^161^ and Cys^90^ residues being 8.2 Å (1 Å=0.1 nm) apart [31]. Ubiquitin vinyl methyl ester (UbVME) is a synthetic ubiquitin substrate containing a rigid extension that mimics the transition state of ubiquitin-substrate hydrolysis, allowing it to bind covalently to the catalytic Cys^90^ cysteine residue in the active site of UCH-L1 (Figure 4) [42]. Crystallographic data indicate that when UCH-L1 is bound to ubiquitin, a conformational change occurs that brings the residues of the catalytic triad into closer proximity and promotes enzymatic activity [43].

### Active-site loop

The UCH class of DUBs all contain an unstructured loop that restricts access to the active site. UCH-L1 contains the shortest loop in the UCH class, which prevents access to the active site for all proteins except for very short disordered peptides (∼10 amino acids) conjugated to ubiquitin [31]. In crystal structures obtained so far, with and without ubiquitin bound, the widest diameter under the active site loop is approximately 10 Å, meaning that any substrate would have to ‘tunnel’ under the loop to allow ubiquitin to dock in the active site (Figure 4). This severely restricts possible UCH-L1 substrates because folded proteins are not able to access the catalytic domain [31,43]. Consistent with this modelling data, *in vitro* assays have shown that UCH-L1 can bind and efficiently hydrolyse ubiquitin-AMC–a ubiquitin molecule conjugated to a small organic fluorescent probe containing two benzene rings [44]–but it cannot bind slightly larger ubiquitin-sepharose conjugates [45]. In contrast, UCH-L3 contains an extended loop that enables it to bind larger ubiquitin-conjugates, such as ubiquitin-sepharose, and peptide sequences up to 80 amino acids in length. It has been reported that UCH-L3 regulates processing of UBA80, a ribosomal-ubiquitin fusion gene [46,47], suggesting that UCH-L1 and UCH-L3 have distinct substrates and functions.

It should be noted, however, that *in vitro* assays have also shown that the efficiency of UCH-L5 (UCH37) at cleaving ubiquitinated substrates can vary enormously depending on the reaction conditions used, suggesting that the simplified assays used so far may not accurately reflect the *in vivo* conditions necessary for UCH-L1 substrate hydrolysis [48,49]. For example, based on UCH-L1 protein structure, it has been hypothesized that the short active site loop adjoins regions of potential flexibility and so could swing out to adopt an extended, accessible conformation, induced by binding the correct substrate [31], although no experimental evidence of this has yet been found.

## FUNCTIONAL ROLES OF UCH-L1

UCH-L1 has a high affinity for ubiquitin, which it can efficiently hydrolyse from small C-terminal extensions in *in vitro* assays [50]. It also has high affinity for monomeric ubiquitin-like molecule NEDD8, but cannot hydrolyse it, unlike the homologous but more widely distributed UCH-L3 [51]. Indeed, the possibility of UCH-L1 exerting an effect through binding to and/or regulating NEDD8 is intriguing because NEDD8 is the most abundant ubiquitin-like molecule (UBL) in neurons [52] and its regulation and roles remain to be fully explored.

### Homeostasis

Lys^48^-polyubiquitin chains are recognized by proteasomal subunits and cofactors allowing the targeting of ubiquitinated substrates for degradation. The ubiquitin molecule itself, however, is recycled. The 19S proteasomal lid contains subunits that can recognize and deubiquitinate substrate proteins, releasing mono- and poly-ubiquitin chains, however it has been proposed that recycled ubiquitin molecules can still be linked to small peptide fragments, which need to be processed for re-use [46,53]. Consistent with a role in ubiquitin processing UCH-L1 can efficiently cleave short disordered peptides of ∼10 residues from the C-terminus of ubiquitin in *in vitro* substrate hydrolysis assays [46]. Furthermore, free monomeric ubiquitin is reduced by 20–30% in the brains of UCH-L1 deficient mice [54–56]. These data strongly suggest that UCH-L1 can trim small disordered peptides from the C-terminus of ubiquitin and increase monomeric ubiquitin levels, consistent with a role in maintaining a pool of available ubiquitin in the cytosol. It should be noted, however, that UCH-L1 exogenously expressed in mouse embryonic fibroblasts (MEFs) binds to and increases free ubiquitin [54]. This also occurs with expression of a hydrolase-deficient C90S mutant, but not a non-ubiquitin binding (D30K) mutant of UCH-L1. These findings have led to the proposal that it is the ability to bind ubiquitin and increase its half-life, rather than the hydrolytic function of UCH-L1 that mediates the increase in free ubiquitin [57].

### Proteasomal function

Although UCH-L1 has been proposed as crucial for maintaining proper proteasomal function [54,58], proteasomal activity is not obviously impaired in UCH-L1 deficient mice [55–57]. Furthermore, unlike UCH-L5, UCH-L1 is not immunoprecipitated with components of the 19S proteasomal lid [56,59].

### Lysosomal function

UCH-L1 deficient nm3419 mice show increased mRNA levels of the lysosomal enzymes cathepsins D and L [56]. Down-regulation of UCH-L1 also correlates with increased apoptosis in fibroblasts from patients with lysosomal storage disorder, although a specific link was not established [60]. However, these phenotypes are not seen in UCH-L1 deficient animal models. Therefore, it remains an open question whether UCH-L1 may regulate a pool of ubiquitin involved in lysosomal trafficking.

### Proubiquitin processing

Monomeric ubiquitin is processed from proubiquitin precursor proteins. Four different genes encode mammalian proubiquitin precursors, two of which are synthesized as ribosomal subunit-fusion proteins and two as polyubiquitin precursors [61]. In bacterial expression assays UCH-L1 does not efficiently cleave monoubiquitin from either ribosomal proteins or polyubiquitin precursors because the folded substrates are too large to fit through the active site loop [46,62]. Intriguingly, however, although UCH-L1 still could not efficiently hydrolyse ubiquitin from ribosomal fusion proteins, it could cleave ubiquitin when co-transfected with a plasmid expressing a polyubiquitin gene, whereas the opposite was true of UCH-L3, suggesting that UCH-L1 may act co-translationally, but not post-translationally, on poly-ubiquitin gene products.

Indeed, although there is clearly a great deal to discover about the mechanisms underlying ubiquitin processing, work using rabbit reticulocytes, mouse liver and HeLa cells has provided evidence that polyubiquitin gene products can be co-translationally processed [47]. UCH-L3 was implicated in these screens, and although UCH-L1 was not identified this could be attributable to the fact that the systems used probably do not express UCH-L1 in sufficient amounts to be detected. Thus in neurons, where UCH-L3 is less abundant, UCH-L1 may fulfil this function. In this scenario as the nascent ubiquitin polypeptide leaves the ribosome it could fit through the restricted active site loop of UCH-L1 and be cleaved allowing full folding into a ubiquitin molecule [46]. Moreover, this mechanism is consistent with the changes to free monoubiquitin levels associated with the gain or loss of UCH-L1 in cells [54,63].

### UCH-L1 deubiquitinase (DUB) activity

UCH-L1 has been proposed to deubiquitinate several exogenously expressed proteins in clonal cell lines, including NOXA and NOX4 [63,64]. Nonetheless, the spatial constraints that limit access of folded proteins to the catalytic site of UCH-L1 make it difficult to understand how it can have general DUB activity. Recent recombinant *in vitro* experiments using UCH DUBs and ubiquitinated substrates show that UCH-L1 is far less efficient than the homologous UCH-L3 [12]. Overall, current data suggest that direct substrates for DUB activity of UCH-L1 cannot be fully folded ubiquitinated substrates. Rather, as outlined above, UCH-L1 is highly efficient at cleaving monoubiquitin from small disordered peptides covalently linked to the C-terminus of a ubiquitin molecule [45].

DUBs act as part of larger protein complexes and the identification of component proteins provides information about the pathways and functions regulated. As discussed below, proteins reported as UCH-L1 interactors from co-immunoprecipitation studies include amyloid precursor protein (APP) [65] and tubulin [65,66]. A high-throughput, unbiased MS screen of the human DUB interactome in cell lines detected an interaction between UCH-L1 and two keratin proteins as well as the uncharacterized coiled-coil domain-containing protein 14 (CCDC14) [30]. Notwithstanding these findings, few functionally verified interaction partners of UCH-L1 in the brain have yet been identified. Nonetheless, we expect that future proteomic analysis of UCH-L1 in neurons will reveal a wide array of novel interactors leading to a much greater understanding of tissue-specific UCH-L1 function.

### Does UCH-L1 have dimerization-dependent E4 ubiquitin ligase activity?

In addition to its monomeric DUB function, a dimeric form of UCH-L1 has been proposed to have ubiquitin E4 ligase function, acting to extend polyubiquitin chains on substrate proteins such as α-synuclein and tubulin [25,66]. Interestingly, the first attempts at producing a crystal structure of UCH-L1 found that the protein existed as an asymmetrical dimer in the crystals, with the two units interacting via a 161° rotation [31]. However, sedimentation equilibrium experiments, performed using the same preparation techniques, detected only a monomeric form, leading the authors to conclude that UCH-L1 does not exist as dimers in solution [31].

It has also been reported that UCH-L1 acts as a ligase to extend Lys^63^ polyubiquitin chains on α-synuclein thereby preventing its proteasomal degradation [25]. However, from the current understanding of UCH-L1 structure, it is unclear how UCH-L1 could extend a polyubiquitin chain on a substrate protein and then have a folded ubiquitin molecule or the substrate pass back through the active site loop. Moreover, subsequent attempts have been unable to recapitulate these results [67].

## UCH-L1 IS NECESSARY FOR AXONAL MAINTENANCE IN THE CNS

Two naturally spontaneously occurring *Uchl1* mutant mice lines and an *Uchl1* knockout mouse have been characterized [55,56,68]. The phenotypes of all three are remarkably consistent and suggest that UCH-L1 has a crucial role in the maintenance of axonal health and stability.

### UCH-deficient mouse models

#### The *gad* mouse

The recessive gracile axonal dystrophy (*gad*) phenotype developed spontaneously in a strain of lab mice, leading to sensory ataxia at approximately 3 months, and motor ataxia at 4 months, manifesting first as a hind limb paralysis and followed by death at approximately 6 months [69]. The defect was mapped to an in-frame deletion including exons 7 and 8 from the *Uchl1* gene, corresponding to the loss of 42 residues from 154 aa to 196 aa, including the catalytic His^161^ [55]. Although mRNA transcripts are produced in equivalent amounts to WT (wild-type), there is no UCH-L1 protein, which combined with the recessive nature of the phenotype, suggests that defects in the *gad* mouse are due to UCH-L1 ablation [55]. Post-mortem analysis of homozygous *gad* mice revealed inclusion bodies in axon nerve terminals in the gracile tract of the spinal cord. Axons from dorsal root ganglion cells that pass through the gracile tract possess the longest axons in the mammalian CNS [70]. The affected neurons display spheroid bodies characteristic of a failure of axonal transport and an axonal ‘dying-back’ phenotype, characteristic of ‘Wallerian’ degeneration, a programmed event analogous to, but distinct from, apoptosis [71–73]. Other sensory and motor neurons that possess long axons are also affected and the extent of degeneration is proportional to axon length. The spheroid bodies contain accumulations of amyloid-β (Aβ) protein as well as ubiquitin-positive deposits and the neurons are depleted of free ubiquitin [54,74].

#### The nm3419 mouse

Another spontaneous mutation arose in a separate strain of lab mice, with homozygous mice displaying signs of motor ataxia at ∼1 month and death at ∼6 months [56]. This mutation inserts a premature stop codon that truncates the last 78 amino acids of UCH-L1 although, as with the *gad* mouse, no UCH-L1 protein can be detected [56]. Also similar to *gad* mice, free monomeric ubiquitin is reduced by ∼30% compared with WT mice. Even at pre-symptomatic stages, nm3419 mice corticospinal motor neurons show increased ER stress that correlates with disintegration of the apical dendrite and spine loss [75].

#### The *Uchl1* knockout mouse

A specific UCH-L1^−/−^ mouse has been generated that displays a similar ataxic phenotype of progressive paralysis and death at ∼7 months [68]. UCH-L1 ablation resulted in the degeneration of presynaptic terminals at the neuromuscular junction, a loss of synaptic vesicles and the presence of tubulo-vesicular structures comparable to those seen in dynamin-1 null mice [76].

Taken together the results from mouse models indicate that, while not essential for neuronal development, UCH-L1 is absolutely required for the maintenance of axonal integrity.

## UCH-L1 AND DISEASE

Cells have developed numerous mechanisms to deal with misfolded or aberrant proteins, mostly involving ubiquitin-mediated degradation pathways, including the formation of aggresomes and initiation of autophagic pathways [77]. However, when these processes stall or become overwhelmed, as occurs in neurons under prolonged stress conditions, they can contribute to the pathogenesis of disease [78,79]. The protein aggregates and inclusions that arise in many neurodegenerative diseases are ubiquitin-rich because the aggregated proteins have been ubiquitinated and marked for destruction [80,81]. Depending on the circumstances, UCH-L1 has been proposed to constitute part of the cell's survival response or have a direct role in disease progression [81].

### Human *Uchl1* mutation

Recently a Glu7Ala point mutation in UCH-L1 was identified as the cause of early onset neurodegeneration in three siblings who appeared normal at birth, but became blind at 5 years old and suffered progressive neurological dysfunction and cerebellar ataxia, and were unable to stand by the age of 30 [67]. No phenotype was seen outside of the nervous system. The Glu^7^ residue in UCH-L1 is required for ubiquitin binding [31], and *in vitro* assays with a Glu^7^ mutant show an almost total abolition of Ub-AMC hydrolysis compared with WT [67]. The ataxic phenotype observed in humans expressing ubiquitin binding/hydrolysing deficient UCH-L1 suggests that the axonal degeneration observed in the mouse models are probably due to loss of this function. An interesting line of future investigation would be to discover if a homozygous Cys^90^ hydrolase-deficient UCH-L1 mutant could produce a similar phenotype and thus ascertain whether it is the binding or hydrolytic property of UCH-L1 responsible for this effect.

### UCH-L1 oxidative-modification at Cys^152^

A consistent theme of the involvement of UCH-L1 in neurodegenerative diseases is the extensive oxidative modifications that render UCH-L1 susceptible to unfolding and toxic gain-of-function through exposure of the hydrophobic protein core [36,37,82–84]. For example, the oxidative stress products cyclopentenone prostaglandins (CyPGs) and 4-hydroxynonenal (4-HNE) both decrease UCH-L1 solubility and facilitate aberrant protein interactions [85].

More specifically, CyPGs such as 15d-PGJ_2_ are fatty-acid metabolites derived from cyclooxygenase-2 (COX2), induced following ischaemic injury, and are implicated in the pathogenesis of neurological diseases [86,87]. UCH-L1 is covalently modified by 15d-PGJ2, at Cys^152^, a residue that is not present in UCH-L3 [36,88] causing a loss of secondary structure and protein stability [36]. Although Cys^152^ is situated in the short unstructured active site loop (Figure 2C), it has been proposed that 15d-PGJ2 binding acts as a lipophilic wedge to disrupt the tightly packed hydrophobic core leading to destabilization and aggregation. Consistent with this, a C152A knock-in mouse rescued the defects seen in WT mice following CyPG treatment, including reduced cytotoxicity and UCH-L1 protein aggregation, as well as fewer ubiquitinated aggregates in total [89].

### Neurodegenerative diseases

Proteomic screens have indicated that UCH-L1 undergoes oxidative modification in both Alzheimer's disease (AD) and Parkinson's disease (PD). UCH-L1 solubility is decreased by the oxidation of cysteine and methionine residues and carbonyl formation [90–92] and the resultant increase in insoluble UCH-L1 is proportional to the number of tau-immunoreactive tangles [93,94]. The APP/PS1 mouse model of AD, which overproduces Aβ, shows similar redistribution of soluble and insoluble UCH-L1 to that observed in human AD brain, with a reported ∼20% reduction of *in vitro* hydrolytic activity [95]. Mechanistically, it is likely that the shift from soluble to insoluble forms of UCH-L1 and loss of hydrolytic activity is due to oxidative modification disrupting its native structure, making it prone to aggregation [35,36,90].

### UCH-L1 and Parkinson's disease

The loss of dopaminergic neurons in PD is preceded by the formation of Lewy Bodies, insoluble proteinaceous inclusions enriched with ubiquitinated aggregates, as well as displaying extensive protein oxidative modification [96]. Most cases of PD are sporadic, although familial strains of the disease exist and a few of these have been matched to specific genomic mutations [97], including UCH-L1.

#### I93M

The I93M point mutation in UCH-L1 has been the focus of considerable research because it occurred in four out of seven family members who developed PD. As a result UCH-L1 has been designated a Parkinson's susceptibility gene and given the alternative name of *PARK5* [98]. It should be noted, however, that no effect was seen in the presumed carrier of the mutation and these observations do not satisfy the formal criteria for a genetic linkage [16,98].

Transgenic mice expressing the human I93M gene are born normally and are fertile [84]. However, they do display aberrant dopaminergic neuron morphology in the substantia nigra at 12 weeks, consistent with degeneration and a loss of dopaminergic neurons at 20 weeks [84]. This is unlikely to be due to loss of UCH-L1 hydrolytic activity since heterozygous *gad* mice are healthy and UCH-L1-deficient mice do not develop Parkinsonian symptoms. Rather, I93M likely gives rise to a dominant toxic gain-of-function, so studies have focused on the physical properties of the mutant protein. The Ile^93^ site is located in an intramolecular α-helix near the active site and contacts the hydrophobic core of UCH-L1 (Figure 1). The I93M mutation decreases UCH-L1 solubility, corresponding with an apparent loss of α-helical structure seen via circular dichroism, and a reduction in hydrolytic activity by approximately 50% [37,65,99]. Thus, it has been proposed that the I93M mutant behaves similarly to oxidatively modified forms of UCH-L1 [65]. However, another study using NMR reported that the I93M mutant is well folded and structurally similar to the wild-type protein, with only minor disturbance around the site of mutation [36].

#### S18Y

By contrast, an S18Y mutation in UCH-L1 has been reported to exert a neuroprotective effect against PD [100]. The S18Y mutant was initially reported as a polymorphism, present in approximately 46–61% of those studied in Asian populations, and 16–24% in European Caucasian populations who show a reduced risk of PD [100]. Multiple subsequent studies have yielded contrasting results and the findings have been vigorously contested. A meta-analysis concluded that although there was moderate basis for protection within the separate Asian and Caucasian populations, where the effect was reported as being recessive or dominant respectively, the effects seen were contradictory and as a whole there was no significance [101]. At a protein level, the Ser^18^ side chain does not affect UCH-L1 structure or ubiquitin binding [29] suggesting that any protective actions likely arise from altered protein–protein interactions at, or near this site.

### UCH-L1 in spontaneous PD

UCH-L1 is covalently modified by the endogenous parkinsonism-inducing dopamine derivative 1-(3′,4′-dihydroxybenzyl)-1,2,3,4-tetrahydroisoquinoline (3′,4′-DHBnTIQ), suggesting a possible role in the pathogenesis of idiopathic PD [102]. Moreover, like CyPGs (see above), 3′,4′-DHBnTIQ binds UCH-L1 specifically at Cys^152^ *in vitro*. This increases the amount of insoluble UCH-L1 and reduces its hydrolase activity in SH-SY5Y cells. These results are consistent with the conserved Cys^152^ being a site of modification with the potential to disrupt UCH-L1 stability, leading to neuronal cell death [102]. Although further investigation is required, the data point to a possible mechanistic explanation for how UCH-L1 could misfold and form protein aggregates selectively in dopaminergic neurons in idiopathic forms of PD.

### UCH-L1 as an antioxidant

The complex morphology of neurons dictates a high membrane-to-cytoplasm ratio and synapses require a high proportion of unsaturated fatty acids that regulate membrane fluidity [103]. However, unsaturated fatty acids are susceptible to lipid peroxidation [104], suggesting that neurons require additional mechanisms to regulate lipid metabolism and contain oxidative damage. One possible explanation for the limited deubiquitinase activity of UCH-L1 is that it fulfils other key roles independent of any DUB activity, and, although full mechanistic data are yet to be provided, UCH-L1 has been proposed as a neuronal antioxidant [81,96]. This role could explain the presence of insoluble or misfolded UCH-L1 in many neurodegenerative diseases [90,105]. One hypothesis is that the conserved Cys^152^ residue (see above) acts as a redox buffer in neurons and reacts with, and chelates, free radicals to maintain short-term cellular function [90].

Consistent with this, N2a cells treated with antisense UCH-L1 cDNA to down-regulate UCH-L1 expression were more susceptible to oxygen/glucose deprivation (OGD) induced cell death [106]. Moreover, *gad* mice show increased vulnerability to lipid peroxidation, and damage is further increased in neurons cultured in media deficient in Vitamin-E (α-tocopherol), which is an antioxidant that protects cells from ROS (reactive oxygen species) damage. This is particularly relevant because chronic Vitamin-E deficiency causes gracile tract degeneration, similar to UCH-L1 deficient mouse models [107–109].

Overall, this hypothesis suggests that the abundance and diffuse cytoplasmic distribution of UCH-L1 allows for the chelation of excess ROS during acute damage, enabling the cell to continue to function in the short term at the expense of ubiquitin homeostasis.

## CONCLUSIONS AND FUTURE DIRECTIONS

### What does UCH-L1 do?

Despite intense research efforts the precise functions of UCH-L1 remain enigmatic. However, recent progress in defining the folding and tertiary structure has provided new insights. UCH-L1 has high affinity for monomeric ubiquitin, but is a poor hydrolase of ubiquitinated proteins due to restricted access to the active site [31]. Thus, evidence for UCH-L1 as an ubiquitin processing enzyme is much more compelling than evidence that it deubiquitinates substrate proteins. Furthermore, the fact that UCH-L1 can process short disordered peptide sequences, suggests a role in regulating particular forms of ubiquitin homeostasis. Nonetheless, it is still unclear whether the effects observed on monoubiquitin levels are simply due to ubiquitin binding by UCH-L1 or whether hydrolytic activity is required.

### UCH-L1 and maintenance of axonal integrity

UCH-L1 is necessary for the maintenance of axonal health and stability and its loss results in axonal degeneration and neuronal death. However, despite this clear and reproducible phenotype, as set about above, the mechanisms underlying this degeneration are unclear. We expect that future experiments exploring whether the Cys^90^ mutation causes the same axonal degeneration and ataxic phenotype as the Glu7Ala mutation *in vivo* will help resolve these outstanding questions.

Motor neurons may be particularly susceptible to UCH-L1 loss because they contain a specific ubiquitin pool or pathway that requires UCH-L1 regulation. Also, the high energy and protein turnover burdens required to maintain extensive axonal projections mean that they operate very close to their maximum capacity and that they are more vulnerable to defects that other neuronal types can withstand for longer. It is also possible that UCH-L1 may regulate axon maintenance via microtubule-associating proteins that are crucial for both axonal transport and stability [66,85].

### Linking protein instability to neurodegenerative disease

Beyond rare diseases caused by mutations in UCH-L1 that result in axonal degeneration, UCH-L1 is also implicated in other forms of neurodegenerative disease, most notably Parkinson's disease. Recent work highlights how residues in UCH-L1, particularly Cys^152^, are readily modified by oxidation and that this can lead to destabilization of the protein and exposure of the hydrophobic core, which results in cytotoxic gain-of-function of insoluble UCH-L1. Intriguingly, oxidative damaged induced instability and aggregation is prevented by Cys152Ala mutation, which presents an exciting possibility for therapeutic intervention.

Overall, although UCH-L1 retains some mystique there has been significant progress towards defining its roles in healthy and diseased neurons. We anticipate that in the next few years a more complete understanding will lead to new strategies to exploit its potential as a therapeutic target.

## Abbreviations

Aβ: amyloid-β
AD: Alzheimer's disease
APP: amyloid precursor protein
CyPG: cyclopentenone prostaglandin
3′,4′-DHBnTIQ: 1-(3′,4′-dihydroxybenzyl)-1,2,3,4-tetrahydroisoquinoline
DUB: deubiquitinase
PD: Parkinson's disease
ROS: reactive oxygen species
UbVME: ubiquitin vinyl methyl ester
UCH: ubiquitin C-terminal hydrolase
UCH-L1: ubiquitin C-terminal hydrolase L1
